# Peroral Toxicological Assessment of Two-Dimensional Forms of Nickel Nanoparticles Sized between 20 and 120 nm

**DOI:** 10.3390/nano12193523

**Published:** 2022-10-08

**Authors:** Vladimir A. Shipelin, Antonina A. Shumakova, Eleonora N. Trushina, Oksana K. Mustafina, Alexander G. Masyutin, Alexey I. Kolobanov, Ilya E. Sokolov, Ivan V. Gmoshinski, Sergey A. Khotimchenko, Dmitry B. Nikityuk

**Affiliations:** 1 Laboratory of Food Toxicology and Safety Assessment of Nanotechnology, Federal Research Centre of Nutrition, Biotechnology and Food Safety, 109240 Moscow, Russia; 2 Academic Department of Innovational Materials and Technologies Chemistry, Plekhanov Russian University of Economics, 117997 Moscow, Russia; 3 Laboratory of Immunology, Federal Research Centre of Nutrition, Biotechnology and Food Safety, 109240 Moscow, Russia; 4 Department of Biology, Lomonosov Moscow State University, 119991 Moscow, Russia; 5 Department of Food Hygiene and Toxicology, I.M. Sechenov First Moscow State Medical University (Sechenov University), 119991 Moscow, Russia; 6 Laboratory of Sports Anthropology and Nutriciology, Federal Research Centre of Nutrition, Biotechnology and Food Safety, 109240 Moscow, Russia; 7 Department of Operative Surgery and Topographic Anatomy, I.M. Sechenov First Moscow State Medical University (Sechenov University), 119991 Moscow, Russia

**Keywords:** nickel, nanoparticles, oral toxicity, acute toxicity, rats, behavioral responses, neurotoxicity, apoptosis

## Abstract

Nickel (Ni) nanoparticles (NPs) are used as technological aids–catalysts in the oil and fat industry, in pharmaceuticals, and in the production of cosmetics and pesticides. The acute and subchronic oral toxicity of metallic Ni in the nanoform is not well understood. The study aimed to investigate the acute and subchronic oral toxicity of Ni NPs to rats. We used two NP preparations (Ni NP1 and Ni NP2) with spherical particles and an average diameter of 53.7 and 70.9 nm according to the electron microscopy data. In the study of acute toxicity, both kinds of Ni NPs were administered to male and female Wistar rats aged 8 weeks as a single dose of 2000 mg/kg b.w. through a gastric gavage. In the subchronic experiment, male Wistar rats initially aged 7 weeks received for 92 days Ni NP1 and Ni NP2 as well as the “traditional” soluble salt form of Ni (Ni basic carbonate) at doses of 0.1, 1, and 10 mg/kg body weight (mg/kg b.w.) in terms of Ni content as a part of the diet consumed. As a result, in an acute study, the oral LD_50_ for Ni NP2 in male and female rats was about 1600 mg/kg b.w. (IV hazard class). The oral dose of Ni NP1 equal to 2000 mg/kg b.w. exceeded LD_100_ for males and corresponded to LD_90_ for females. In the subchronic study, the bioaccumulation of both Ni NPs as well as Ni salt was observed in the kidney but not in the liver and spleen. Ni NP1 decreased body weight only at a dose of 1 mg/kg b.w.; affected the relative weight of the spleen at 0.1 mg/kg, the brain at 1.0 mg/kg, and the thymus at 10 mg/kg; and decreased locomotor activity at 0.1 and 10 mg/kg. Thus, for Ni NP1, in such cases where a monotonic dose–response relationship could be traced, LOEL could be stated at 10 mg/kg b.w./day for 92 days of oral intake. However, for some endpoints where such a monotonic relationship could be absent, significant toxic effects were observed even at a dose 0.1 mg/kg. In the case of Ni NP2, changes in the relative weight of the liver, thymus, and brain were recorded starting from 0.1 mg/kg b.w.; locomotor activity decreased starting from 0.1 mg/kg. Other effects, including basophiles count and platelet system indexes, were observed at a dose of 1 mg/kg or higher. Thus, the LOEL value for Ni NP2 can be fixed at 0.1 mg/kg. The critical organs affected by both Ni NPs were the brain and immune system. Most of the toxic effects exhibited by metallic Ni NPs were absent or had an opposite orientation upon administration of equivalent doses of Ni in the salt form which indicates the signs of “nanotoxicity” in metallic Ni NPs. In conclusion, the data obtained show that there may be some additional health risks caused by the intake of Ni in a nanoform compared to soluble ionized forms of this element at equivalent doses.

## 1. Introduction

Nickel (Ni) nanoparticles (NPs) are used in the food industry as a catalyst in the hydrogenation of fats [1]. Due to the small size of NPs, hydrogenation reactions with their participation occur under more equilibrium conditions than in the case of using the catalysts of a traditional structure, and the side formation of unwanted by-products such as trans fatty acid isomers are reduced [2]. Several highly efficient catalysts based on Ni NPs immobilized on inert supports have been developed with sizes in the nanoscale [3], proposed both for the oil and fat industry and for processes in fine organic synthesis, including pharmaceutical applications [4]. In medicine, Ni NPs are used in controlled magnetic hyperthermic therapy and theranostics [5]. Ni NPs can also be present in cosmetics, including foundations and dyes [6]. It was proposed to use the NPs of Ni and its compounds obtained by biotechnology as insecticides [7]. The annual production of Ni-containing NPs only in the USA was estimated in 2019 at 20 tons and tends to further increase [8].

The above circumstances indicate that soon a significant increase in human exposure to Ni compounds in nanoform should be expected [9]. This creates potential health risks due to the high toxicity, possible carcinogenicity [10], and allergenicity [11] of Ni and its compounds for humans. Despite this, many factors that determine the risks of Ni-containing NPs remain poorly understood. The toxicity of Ni NPs, its oxide (II), and hydroxide were characterized in the most detail after intrapharyngeal and inhalation administration, which reflects the opinion that exposure to these nanomaterials through inhalation predominates as an occupational factor in the workplace [12]. At the same time, the possible consequences of oral intake of Ni NPs and their compounds have been insufficiently studied. Although, the oral route of exposure should be borne in mind due to the possible presence of residual amounts of Ni-containing catalysts in food products, as well as its contamination with Ni NPs from other above-mentioned sources. According to [13], Ni oxide NPs have insignificant acute oral toxicity for rats (LD_50_ > 2000 mg/kg body weight). However, subchronic oral administration revealed a number of pathological effects associated with organotoxicity and the development of oxidative stress [14]. At the same time, there are very few data in the available literature on the oral toxicity of the NPs of metallic Ni. In addition, the extent to which particle size affects the toxicity of Ni-containing nanomaterials has not been sufficiently studied. Most of the studies presented in the literature on the toxicity of Ni and its oxide NPs in both in vitro and in vivo systems were performed using samples of nanomaterials of a single size. Only a few works indicated that the toxicity of Ni and its compounds in nanoform may be higher compared to particles larger than 100 nm (microparticles) [15,16,17] and even compared to the completely soluble salt form of Ni [18].

The aim of this work is the toxicological characterization of metallic Ni NPs of two different dimensional forms in the study of behavioral responses; integral, hematological parameters; and apoptosis of liver cells in rats.

## 2. Materials and Methods

### 2.1. Nanomaterials

We used two Ni NP preparations manufactured by Nanostructured & Amorphous Materials Inc. (Los Alamos, NM, USA) with articles 0282 HW and 0283 HW (hereinafter referred to as Ni NP1 and Ni NP2, respectively). The diameters of the primary NPs of metallic Ni as declared by the manufacturer should be equal to 20 nm and 40 nm. The purity of the preparations, according to the manufacturer’s information, was 99.5% for Ni, the true density was 8.908 g/cm^3^, and the content of impurities (in weight %) was as follows: C ≤ 0.180 (Ni NP1) and ≤0.08 (Ni NP2), Fe ≤ 0.008 (Ni NP1) and ≤0.08 (Ni NP2), Na ≤ 0.0002, Pb ≤ 0.006 (Ni NP1) and ≤0.001 (Ni NP2), As ≤ 0.001, Cu ≤ 0.01, Mn ≤ 0.02. The first of the preparations was supplied in the form of a paste moistened with water, and the second was a powder, dispersible in water with the formation of stable black suspensions. Ni carbonate basic NiCO_3_ × Ni(OH)_2_ × nH_2_O of “pure” qualification was purchased in LLC “Plasmoterm” (Schelkovo, Moscow district, Russia) with 45% Ni content used as a source of the “traditional” salt form of Ni.

### 2.2. Animals and Design

During the experiments, we used male and female Wistar outbred rats obtained from the Stolbovaya nursery of the Federal State Budgetary Institution Scientific Center for Biomedical Technologies of the Federal Medical and Biological Agency of Russia. The work with animals was carried out in accordance with the rules of good laboratory practice and international recommendations for the humane treatment of animals [19]. The design of the experiment was approved by the Ethics Committee of the Federal Research Centre of Nutrition, Biotechnology and Food Safety (protocol No. 7 of 17 September 2021). The study of Ni NPs’ acute toxicity was carried out according to official OECD guidelines [20]. The experiment was performed on two groups of females and two groups of male rats, aged 7 weeks, with 8 individuals each with an initial average body weight (b.w.) of 236 ± 10 and 203 ± 10 g, respectively. All animals received Ni NP1 or Ni NP2 once intragastrically at a dose of 2000 mg/kg b.w. The survival rate and morbidity of the animals were observed during the first six hours after administration, with an interval of one hour and then daily; body weight was determined once every two days. The dead animals were subjected to an overview pathological examination. The surviving animals were removed from the experiment on the 14th day by CO_2_ inhalation, and the pathology of the internal organs was studied during the necropsy. LD50 values were estimated from mortality data for 14 days by linear extrapolation in semi-logarithmic coordinates.

**Figure 1:**
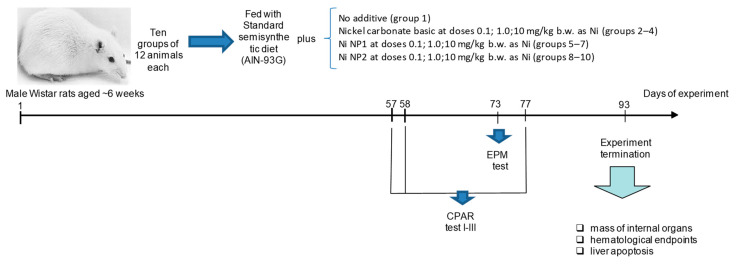
Schematic representation of the experimental design of subchronic toxicity study.

A subchronic 92-day study was performed on 120 male rats with an initial b.w. 180 ± 10 g aged 6 weeks at the beginning of the study. After one week of adaptation, the animals were randomly divided into 10 groups of 12 individuals. Initial body weight did not differ between groups (*p* < 0.05, ANOVA). Animals were housed in groups of two in plastic cages on sawdust bedding. The first (control) group of rats received a balanced semi-synthetic diet according to AIN-93G [21] and reverse osmosis drinking water without restrictions. Groups 2 to 4 received the same diet and the “traditional” salt form of Ni at calculated doses of 0.1, 1.0, and 10 mg/kg b.w. in terms of Ni in the composition of the consumed feed. The composition of the diet of rats of groups 5 through 7 was supplemented with the Ni NP1 preparation in doses equivalent to groups 2 through 4 based on Ni, respectively, and in groups from 8 through 10 with Ni NP2 in the same amounts. To maintain a constant daily dose of Ni, the weight of feed consumed was recorded daily, adjusting, if necessary, the amount of added Ni-containing preparations. During the preparation of the diets, Ni NPs were added in the form of an aqueous suspension after ultrasonic treatment for 15 min at a frequency of 44 kHz and specific power of 2 W/cm^3^ with ice cooling. The average calculated Ni consumption in the composition of the basic diet (AIN-93G) in the control group was 0.03 mg/kg b.w. A schematic representation of the experimental design is shown in Figure 1.

During the experiment, the general condition of animals was assessed daily; body weights were determined at weekly intervals. The feed residues that were not consumed by animals were weighed daily and the actually administered doses of all forms of Ni were calculated. The amounts of Ni NPs and Ni salt added to the diets were corrected when it was necessary to maintain the given doses of Ni.

Animals were removed from the experiment on the 93rd day after 16-h fasting by decapitation under ether anesthesia. Blood samples were collected from the vena cava inferior in tubes using an anticoagulant (EDTA tripotassium salt) to determine hematological parameters. Organs (liver, kidneys, spleen, lungs, heart, adrenal glands, thymus, gonads, and brain) were isolated with sterile surgical instruments and their weight was determined on an electronic balance with an error of ±1 mg.

### 2.3. Behavioral Responses

On days 57, 58, and 77 of the experiment, cognitive function was assessed using the “Conditioned reflex of passive avoidance” test (CRPA); on the 73rd day, the level of anxiety-like functions and motor (locomotor) activity were assessed in the “Elevated plus maze” (EPM) test in installations manufactured by “Panlab Harvard Apparatus” (Barcelona, Spain). The testing methodology was described in detail in [22].

### 2.4. Methods of Sample Analysis

The characterization of nanosized forms of Ni was performed by transmission electron microscopy (TEM) on a JEM 2100 instrument (Jeol Ltd., Musashino, Akishima, Tokyo, Japan) at an accelerating voltage of 80 kV. The analysis was carried out in an aqueous dispersion of NPs of both types, and dried on the surface of copper grids covered with carbon. The qualitative elemental composition of NPs was studied using the built-in option of energy-dispersive microspectrometry (EDS). The plotting of the particle size distribution was performed after determining the diameter of 100 particles of each type on microphotographs.

Zeta potential measurements were assessed by electrophoretic light scattering (ELS) using a Nano Zeta potential analyzer (DelsaNano C Submicron, Beckman Coulter Delsa™, Krefeld, Germany). Each sample was properly diluted using Milli-Q water as the diluent with ultrasonic treatment. Measurements were performed in a Flow Cell (Beckman Coulter Delsa™) at a temperature of 25 °C and measuring the angle of 15°. The zeta potential was extracted from the Helmholtz–Smoluchowski equation. Those values were listed as means of triplicate runs per sample. The data were processed via the Delsa Nano UI 3.73 software.

Hematological parameters (erythrocyte, leukocyte, and platelet counts) were studied on a Coulter AC TTM 5 diff OV hematological analyzer (Beckman Coulter Life Sciences Co., Indianapolis, IN, USA) with a standard set of reagents (Beckman Coulter France SAS, Villepinte, Roissy, CDG Cedex, France).

The content of Ni in organs (liver, kidney, and spleen) and the purity of the nanomaterials used were determined by inductively coupled plasma mass spectrometry on a 7700x series instrument manufactured by Agilent Technologies (Tokyo, Japan). The mineralization of biological samples was performed under the action of concentrated nitric acid and concentrated hydrogen peroxide in the amount of 2.0 and 0.4 mL per gram of tissue, respectively, in a TOPWAVE automated microwave sample preparation system (Analytik Jena GmbH, Jena, Germany). The mineral content was expressed in µg/g tissue (wet weight). The instrumental error of the analysis method was ±25%. The limit of quantitative determination (LOQ) for Ni was equal to 5.10–3 μg/g of tissue.

Apoptosis of liver cells was studied by flow cytometry using the Annexin-V–7-aminoactinomycin D (AnV–7AAD) system. The principle of the method as described in [23], is that at the initial stages of apoptosis, phospholipids that bind AnV are expressed on the plasma membrane of cells (AnV+7AAD-cells); at the later stages, 7AAD penetrates cells with nuclear DNA staining (AnV+7AAD+cells); and in dead (necrotic) cells, AnV receptors in the membrane disappear (AnV–7AAD+cells). Normal living cells are not stained with any of the dyes (AnV–7AAD-cells). The study was carried out using an automatic system for mechanical tissue homogenization BD Medimachine (Becton Dickenson and Company, Franklin Lakes, NJ, USA) and an FC-500 flow cytometer (Beckman Coulter Life Sciences Co., Indianapolis, IN, USA), reagents, and software Cytomics CXP Software (Beckman Coulter Life Sciences Co., Indianapolis, IN, USA) according to the methods of the manufacturer. Fluorescence detection was performed at wavelengths: λ_1_ = 525 nm, λ_2_ = 675 nm.

### 2.5. Statistics

Statistical processing of the results was performed with the determination of the sample mean (M), standard error of the mean (S.E.M.), sample median (Med), and intervals of the change. The hypothesis of heterogeneity in the distribution of values across groups of animals was tested using a 3-factor ANOVA test. The significance of paired differences between groups was established using the 2-sided Student’s *t*-test with Levene’s correction and the nonparametric Mann–Whitney *U*-test. Differences were considered significant at the level of *p* < 0.05. The SPSS 20.0 software package (IBM Corporation, Armonk, NY, USA) was used for calculations.

## 3. Results

### 3.1. Characterization of Nanomaterials

**Figure 2:**
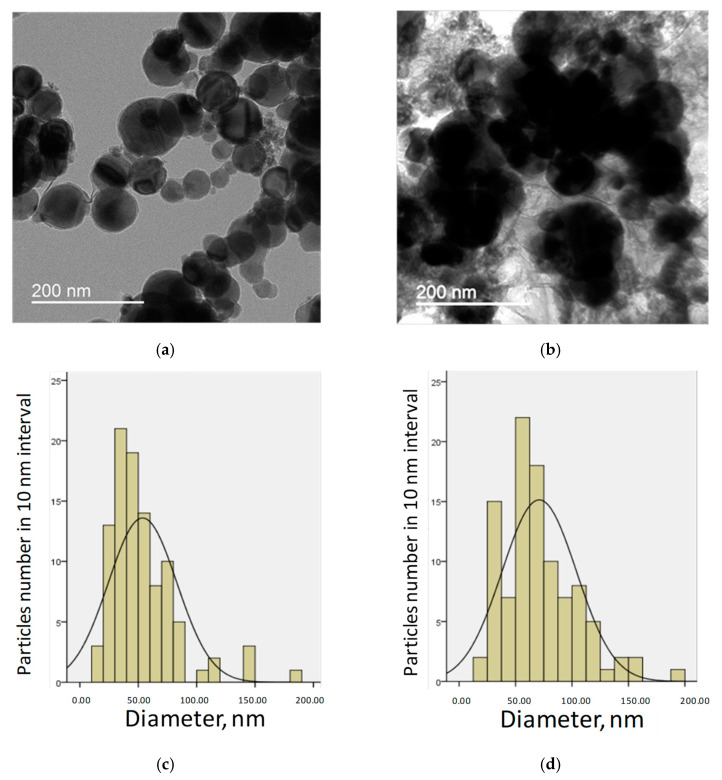
Characterization of metallic Ni NPs of two sizes by TEM. (**a**,**c**)—Ni NP1; (**b**,**d**)—Ni NP2. Representative micrographs (**a**,**b**) and histograms of particle diameter distribution (**c**,**d**).

The results of the characterization of the actual particle diameter distribution of the Ni NP1 and Ni NP2 preparations are presented in Table 1 and Figure 2. As follows from the data in the figure, 55.5% of the particles in the NP1 were less than 50 nm in diameter, 37.8% were from 50 to 100 nm, and only 6.7% of the particles were more than 100 nm in diameter. For NP2, the corresponding number of particles was 24.0%, 57.1%, and 18.9% in the indicated size ranges. The actual average particle diameter of both preparations was equal to 53.7 ± 2.9 nm (M ± S.E.M.) for Ni NP1 and 70.9 ± 3.3 nm for Ni NP2, which was somewhat higher than that indicated in the manufacturer’s documentation (20 and 40 nm, respectively, see Section 2.1). The diameter distribution of Ni NP1 particles was not normal (*p* < 0.05, nonparametric Kolmogorov–Smirnov test); after taking the logarithm of the particle diameter, the distribution acquired a normal form (*p* > 0.1). For Ni NP2, a normal particle diameter distribution was noted (*p* > 0.1). On electron micrographs, the NPs of both preparations had well-defined borders and a rounded shape. The EDS study qualitatively confirmed the dominant presence of Ni in the composition of particles, as well as trace impurity elements such as oxygen (O) and carbon (C), which are apparently associated with partial surface oxidation in an oxygen atmosphere and the transition of a part of Ni oxide to carbonate. According to the data obtained from ICP-MS, in both types of nanoparticles used, the amounts of toxic elements As, Pb, and Cd were ≤0.001% (in weight %).

### 3.2. Acute Oral Toxicity

**Figure 3:**
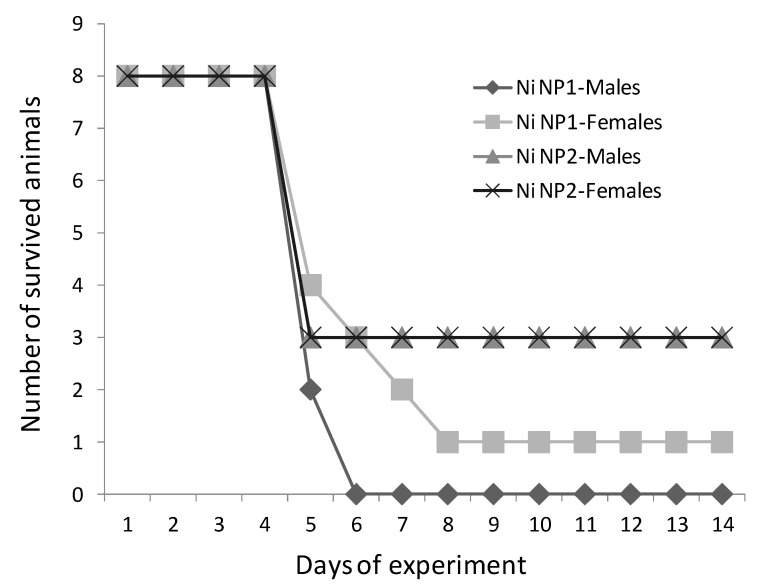
The survival rate of male and female rats within 14 days after a single intragastric administration of Ni NPs at a dose of 2000 mg/kg b.w.

As a result of a single intragastric administration of Ni NP1 to rats, the lethality in the first 14 days in group 1 (females) was 87.5% (7/8), in group 2 (males) it was 100% (8/8). After the introduction of Ni NP2, the lethality in both females (group 3) and males (group 4) was 62.5% (5/8). As follows from the data presented in Figure 3, the death of the main part of the rats occurred within 4–8 days after the administration of both nanomaterials. It was shown at the necropsy that the most probable cause of death of the animals was impaired gastrointestinal motility with the development of obstruction of the small or large intestine. A large amount of black material (presumably, the injected suspension of NPs) was observed to be retained in the stomach, small intestine, cecum, and large intestine; the signs of peritonitis, enteritis, and hepatitis were noted. In contrast to males, in some females, both in group 1 and group 3, the translocation of nanomaterial from the intestine with staining of the peritoneum in black was visually observed postmortem. In the animals that survived by the 14th day, there was no visual nanomaterial in the lumen of the stomach and intestines, and no specific signs of pathology were revealed in the abdominal cavity and chest organs. Thus, for Ni NP1, the dose of 2000 mg/kg exceeded the LD_100_ for males and was close to the LD_90_ for females. For Ni NP2, the calculated LD_50_ value in males and females was about 1600 mg/kg b.w.

### 3.3. Subchronic 92-Day Oral Toxicity

#### 3.3.1. Integral Indicators

**Figure 4:**
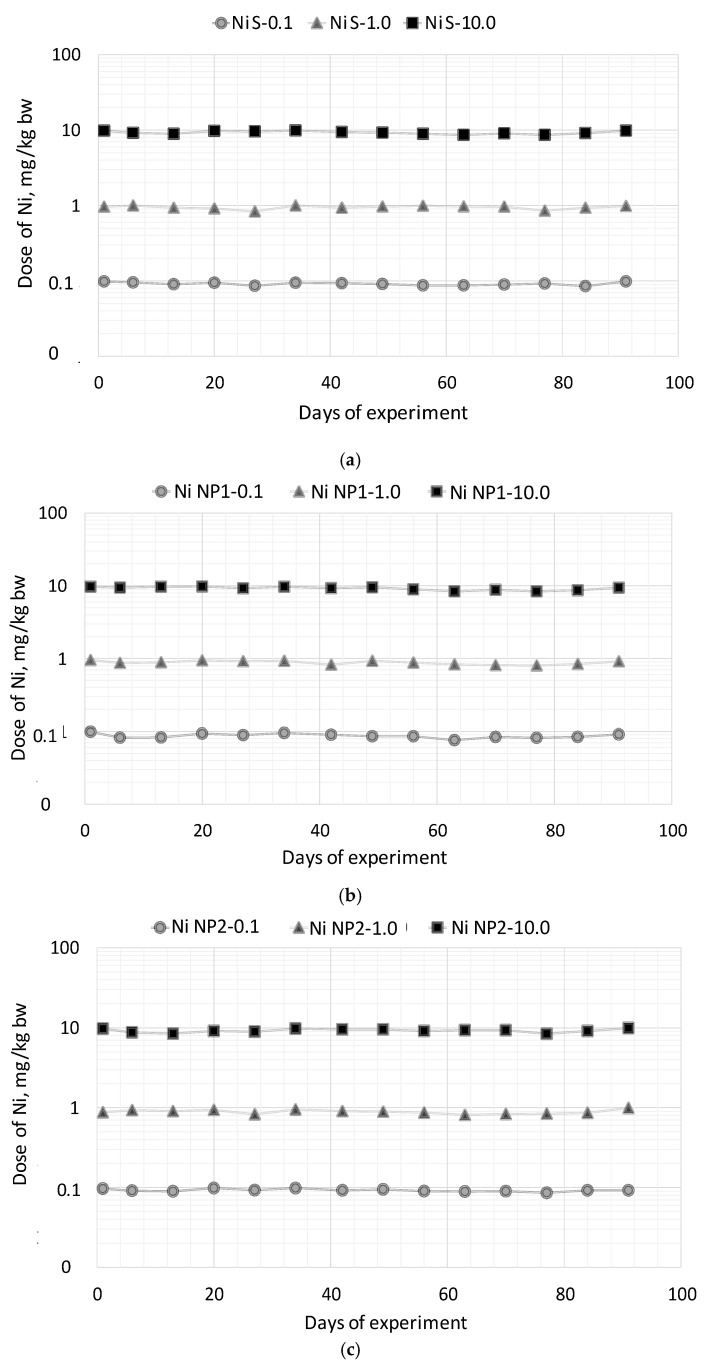
Estimated actual doses of Ni NPs preparations consumed by rats during the 92-day experiment: (**a**) Soluble salt of Ni (Ni S); (**b**) Ni NP1; (**c**) Ni NP2.

**Figure 5:**
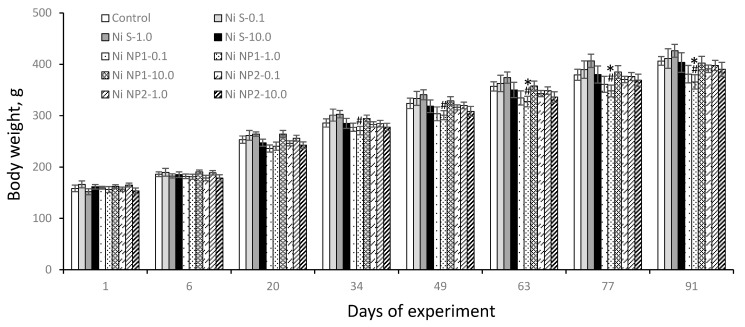
Average body weight of rats (g; M ± S.E.M.) during the experiment. *—the difference with the control group is significant; #—the difference with the group that received the Ni soluble salt (Ni S) in an equivalent dose is significant; *p* < 0.05. The number of animals is 12 in each group.

As follows from the data in Figure 4, throughout the entire course of feeding with experimental diets, the doses of Ni preparations consumed by the rats stably corresponded to the preset values of 0.1 mg/kg b.w. for groups 2, 5, and 8; 1 mg/kg b.w.–3, 6, and 9; 10 mg/kg b.w.–4, 7, and 10, respectively, while the total amount of food consumed did not significantly differ between groups. During the experiment, the death of the animals was not observed, there were no external signs of the disease, and rats were monotonically gaining in body weight. At the same time, as can be seen from Figure 5, rats of group 6, which received Ni NP1 at a dose of 1 mg/kg b.w., significantly lagged in body weight from animals that received the same amounts of Ni soluble salt (Ni S) starting from the 20th day of the experiment, and from animals of the control group starting from the 63rd day (*p* < 0.05). The difference in the increase of body weight in other groups with the control group was insignificant.

At withdrawal from the experiment, as follows from the data in Table 2, rats of group 6 (Ni NP1, 1 mg/kg) had a significantly reduced body weight; they also had significantly increased brain weight; in some animals from this group, signs of hydrocephalus were noted at the section. At a minimum dose of Ni NP1, 0.1 mg/kg b.w. (group 5), there was a significantly reduced relative weight of the spleen, and at the maximum dose (10 mg/kg b.w., group 7), there was a reduced weight of the thymus. In rats of all groups that received Ni NP2 (from 8th to 10th), the weights of the liver and thymus were significantly reduced in comparison with the control, and the weights of the brain were significantly increased. The spleen weight in animals of groups 8 and 10 showed a tendency to decrease, significant in the case of comparison with the corresponding groups receiving Ni soluble salt. This element form in the indicated doses did not affect the relative weight of internal organs, except for a decrease in the relative liver weight in group 2 (0.1 mg/kg b.w., according to Ni).

#### 3.3.2. Behavioral Responses

**Figure 6:**
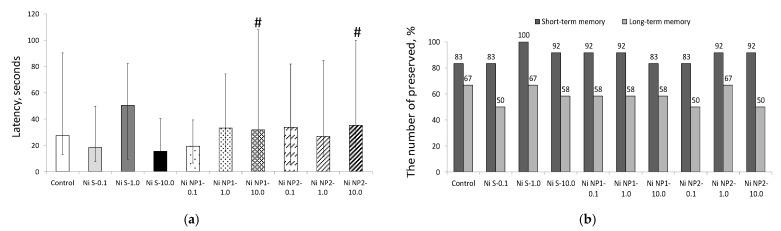
Parameters of rats in the CRPA test: (**a**) latency of entry into the dark chamber, seconds (Med, interval of change); (**b**) the number of animals retaining short-term and long-term memory, %. #—the difference with the group that received the Ni salt in an equivalent dose is significant; *p* < 0.05. The number of animals is 12 in each group.

The study of the cognitive function of rats in the CRPA test (Figure 6) did not reveal significant differences in the degree of preservation of short-term and long-term memory, which varied within 83–100% and 50–67%, respectively, and was within the normal range for animals of this species, gender, and age, as noticed in the variety of previous studies. In rats of group 4 that received Ni salt at a dose of 10 mg/kg b.w., there was a tendency towards a decrease in the latency time of entering the dark compartment of the installation, indicating an increase in anxiety, and the difference with the groups receiving equivalent doses of NPs was significant (*p* < 0.05).

**Figure 7:**
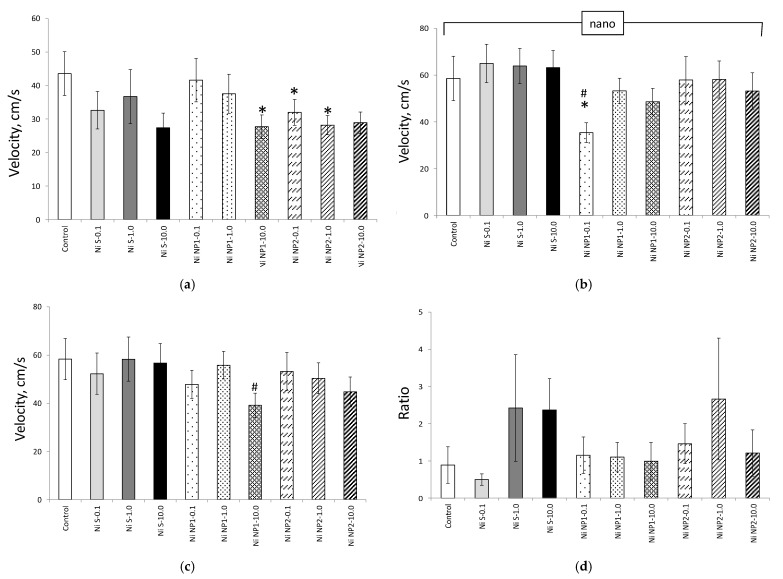
Indicators (M ± S.E.M) of locomotor activity (**a**–**c**) and anxiety (**d**) of rats in the EPM test during the experiment: (**a**) maximum speed in OA, cm/s; (**b**) maximum speed in CA, cm/s; (**c**) maximum speed in general, cm/s; (**d**) the ratio of the residence times in the CA and OA, dimensionless. *—the difference with group 1 (control) is significant; #—the difference with the group receiving the salt form of Ni (Ni S) in an equivalent dose, is significant, Mann–Whitney *U*-test. Horizontal bracket–non-uniform distribution by factors of the presence of nanoform (nano), *p* < 0.05, ANOVA test. The number of animals is 12 in each group.

The influence of Ni NPs on the behavioral responses of animals in the EPM test was manifested mainly in a decrease in the locomotor (searching) activity of animals under the influence of nanomaterials, which was most noticeable in terms of the indicators of the maximum speed developed by rats in the closed (CA) and open (OA) arms of the maze (Figure 7). These changes did not demonstrate a clear dependence on the nanomaterial dose, except for the maximum velocity in the OA in the case of Ni NP1. For the salt form of Ni in equivalent doses, such effects were absent. Anxiety (which is considered the largest at the maximum relative time spent in the CA) was the highest in animals receiving Ni salt (which qualitatively agreed with the CRPA data), as well as Ni NP2 in an intermediate dose. However, the differences in this indicator of the corresponding groups of animals with the control were insignificant.

#### 3.3.3. Hematological Indicators

The consumption of Ni in nanoform at the doses used did not have a significant effect on the indicators of the rat erythrocyte system (data not shown). However, as follows from the data in Table 3, there were various effects associated with the leukocyte count of the blood and the platelet system. Thus, at a dose of Ni NP1 of 10 mg/kg, an increased number of total leukocytes, lymphocytes, and monocytes was observed (the difference was significant *p* < 0.05 in comparison with the group of animals receiving Ni soluble salt). Ni NP2 administered at a low dose (group 8) caused an increase in the levels of total leukocytes; at the intermediate dose (group 9), basophiles; and at a high dose, neutrophyles. At the same time, the increase in the number of monocytes under the influence of Ni NP2 did not occur. Under the influence of Ni NP1 at a low dose, there was a decrease in the average volume of platelets, at a medium dose–in the thrombocrit (*p* < 0.05 compared with the control), and at a high dose, there was a decrease in the total number of platelets. Ni NP2 at a dose of 10 mg/kg caused a decrease in the average platelet volume (significant difference from the control, *p* < 0.05) and caused similar to Ni NP2, but with a less pronounced decrease in the number of platelets.

#### 3.3.4. Liver Apoptosis

As shown by the flow cytometry, both preparations of Ni NPs and the Ni soluble salt in the used dose range did not have a significant effect on the indices of apoptosis of liver cells. The number of living cells by groups of animals was high and varied within 96–97% of their total number, with cells at the “early” stage of apoptosis from 2.6 to 3.1%, and the “late” stage from 0.1 to 0.2%. These values did not significantly differ between the groups of rats and were typical for healthy animals of a given line, sex, and age, i.e., they corresponded to the intra-laboratory norm previously established in a variety of experiments. The number of dead cells (necrosis) in the liver of all animals was very low, not exceeding 0.1%, and did not differ significantly in the groups receiving Ni preparations from the control values.

#### 3.3.5. Nickel Accumulation in the Tissues

As follows from Table 4, the Ni content in the liver of rats receiving Ni soluble salt at a high dose was slightly and insignificantly increased compared to the control (*p* > 0.1). In animals fed with Ni NPs of both kinds, excessive Ni accumulation in the liver was completely absent at all doses. In the kidneys of rats fed with Ni salt, the concentration of the metal significantly increased at intermediate and high doses, while in animals that received both types of Ni NPs it was noticed at a high dose only. At an intermediate dose of Ni, its accumulation in the kidneys after administration of both kinds of NPs was significantly lower than for the equivalent dose of the soluble salt form of Ni. In the spleen of rats, excessive accumulation of Ni was not detected in comparison with the control in all experimental groups.

## 4. Discussion

In the current study, a set of endpoints including behavioral responses; integral, hematological parameters; and apoptosis of liver cells was studied in rats receiving NPs of Ni metal with different dimensional characteristics, in comparison to similar doses of Ni salt, which is completely soluble in the stomach content. The electron microscopy study showed that two preparations of NPs, i.e., NP1 and NP2, were largely different in their size distribution, so the quantity of the particles with a diameter less than 50 nm was 2.3 times more in NP1 than in NP2. Considering the available literature data on the significant size effect on Ni-containing particles’ toxicity in vivo [17], it was of interest to establish possible differences in the toxicological characteristics of NP1 and NP2 between themselves, and in comparison to the salt form of Ni.

In addition to size, a factor affecting the toxicity and bioavailability of NPs may be their aggregation stability. The ζ-potential of the Ni NPs measured by us was +25.71 and −3.33 mV, respectively. This difference in the ζ-potential between the two preparations of NPs may be since one of them was supplied in the form of a powder, and the other in the form of an aqueous paste. As a result, a different number of ionogenic functional groups can be formed on the particle surface. However, this indicates that the stability of particle aggregation in the aqueous dispersion, determined by electrostatic repulsion, is low (the value is in the range from −30 to +30 mV). Concerning NPs included as a part of the diet and in the internal environment of the body, we suggest that electrostatic repulsion does not play a decisive role in particle aggregation, and its degree is determined by the state of the structural–mechanical barrier caused by the adsorption of various biopolymers of the diet and host organism.

As was shown by the experimental data on the single administration of two NPs to male and female rats, these nanomaterials had pronounced acute toxicity (not lower than the 4th hazard class according to [24]), which distinguishes them from the previously studied Ni oxide NPs, which were characterized by very low acute toxicity [13]. On the other hand, the obtained data suggested that subchronic oral administration of Ni NPs at a dose of 10 mg/kg of the body would not be accompanied by lethality in animals. At the same time, the lowest dose studied, i.e., 0.1 mg/kg, is characterized by considerable aggravation compared with the proposed scenarios of oral human exposure to Ni NPs. This is a common approach in biological modeling on small animals that have a high metabolic rate and a shorter lifespan compared to humans. The acute study did not reveal any significant differences between male and female rats that allowed further studies to be carried out only on males.

As follows from the data of the subchronic study, most of the effects caused by Ni NP1 as well as Ni NP2 were not manifested or were manifested to a much lesser extent when animals were exposed to equivalent doses of soluble Ni salt. This may indicate that Ni NPs possess the properties of nanotoxicity in accordance with the definition given recently [25].

The toxicological evaluation of each of the two studied types of nanomaterials should be carried out separately in connection with the data on their physicochemical characteristics, toxicokinetics, and toxicodynamics following the recommendations [26]. According to the data obtained, Ni NP1 showed an effect on the body weight of rats only at a dose of 1 mg/kg b.w. In terms of the relative weight of internal organs, the effect was presented for the spleen only at a dose of 0.1 mg/kg, the brain at 1.0 mg/kg, and the thymus at 10 mg/kg. A decrease in locomotor activity was recorded at 0.1, and 10 mg/kg. The effect on hematological parameters was recorded only at 1 mg/kg in terms of platelet volume fraction. Thus, for Ni NP1, in such cases where a monotonic dose–response relationship could be traced, LOEL was fixed at 10 mg/kg b.w./day for 92 days of oral intake, and the critically affected organs were apparently the brain and the immune system. However, for those endpoints where such a monotonic relationship could be absent, significant toxic effects were observed even at the lowest dose such as 0.1 mg/kg. The latter circumstance may indicate a significant contribution of the aggregation in the biological environment in the manifestation of the effects of this type of NP following [26].

Ni NP2 influenced the relative weight of the internal organs of rats (liver, thymus, brain) at a dose of 0.1 mg/kg b.w. and higher. The level of locomotor activity decreased starting from 0.1 mg/kg but was not statistically significant at the highest dose of 10 mg/kg. Effects on platelet systems have been observed starting at 10 mg/kg; basophiles levels at 1.0 mg/kg (but no dose–response relationship was observed). Thus, the LOEL value for Ni NP2 can be, using the set of studied endpoints, fixed at the level of no more than 0.1 mg/kg. The critical organs of action may be considered as the brain and the immune system. The effect on liver weight was isolated, i.e., not confirmed by the results of the apoptosis study. These results are subject to further refinement using a wider set of biological endpoints, which will be the subject of further study.

The introduction of two 10-fold uncertainty (safety) factors is adopted in toxicological studies on rodents, between which one is associated with the uncertainty in the choice of the exposure scenario and the second with the translation of the results obtained in small animals to humans. Concerning this, it can be concluded that human daily intake of any kinds of Ni NPs in the dimensional range between 20 and 120 nm may not be completely safe at the level of 1 μg/kg of body weight, i.e., 100-fold lower than LOEL fixed in the current study. When comparing these data with the estimated daily intake of Ni (all forms), which is apparently between 74 and 286 μg/day or from 1.1 to 4.1 μg/kg body weight/day for a person weighted 70 kg according to data from multiple sources [27], it can be assumed that there are some additional health risks from the intake of this element with food and water just in a nanoform.

The data in the literature on the oral toxicity of Ni NPs and their oxide are scarce. As a result of a single intragastric administration of NiO NPs sized 16 nm to female rats at a dose of 2000 mg/kg, there was no lethality, but a decrease in the locomotor activity of animals, signs of DNA damage in the liver and kidneys according to the micronucleus and comet tests, and an imbalance of antioxidant defense enzymes [13]. In the 28-day intragastric administration to Wistar rats, NPs of NiO with 13 nm in diameter showed subchronic toxicity at a dose from 50 to 200 mg/kg b.w. [14]. The identified endpoints were histopathological changes in internal organs, an increase in transaminase activity in liver and kidney homogenates, a decrease in superoxide dismutase activity and an increase in catalase activity, depletion of reduced glutathione reserves, and an increase in the level of lipoperoxides. Intragastric administration of NiO NPs sized 50 nm to male Wistar rats for 7 or 14 days at doses from 1 to 4 mg/kg b.w. led to a significant increase in the number of chromosome aberrations, micronuclei, DNA damage, generation of reactive oxygen species, and mitochondrial dysfunction in the liver tissue [28]. An imbalance of antioxidant enzymes and histological changes were noted in the liver. In the only study of subchronic oral toxicity of Ni metallic NPs sized 90 nm in male and female rats at doses of 5–45 mg/kg b.w., within 10 weeks ultrastructural changes in the ovaries and testes, the development of oxidative stress, and the expression of proteins associated with apoptosis have been revealed [29,30]. Thus, as follows from the literature data, the present study showed for the first time the presence of oral toxicity of metallic Ni NPs administered to animals at low doses such as 0.1–1.0 mg/kg b.w.

There were two regularities common to all the revealed effects. First, the effects inherent in Ni NPs were not manifested or manifested themselves completely differently for the soluble salt form of the element in a comparable dose, which could be considered as a specific manifestation of the “nanotoxicity” of substances with a dimensional parameter in the nanoscale [31]. The presence of Ni NPs’ nanotoxicity is indicated by data obtained in cellular tests in vitro where the ability of the salt form of Ni to have a damaging effect on cells was absent [32] or less pronounced [18] compared to Ni NPs in equivalent doses. Second, the two size forms of Ni NPs were characterized by different critical variable parameters, with the exception, in general, of the effect on the brain and behavioral responses. The latter is consistent with the manifestation of neurotoxicity in Ni-containing NPs in vitro [33] and in vivo [34], which depends on the size and composition of particles. At the same time, some signs of increased anxiety in animals were revealed in the case of the soluble salt form of Ni administration in contrast to NPs.

The reasons for the specific effects of nanotoxicity of metallic Ni particles should be discussed in light of the processes occurring on the surface of Ni NPs during their interaction with the biological environment, including the catalytic generation of ROS [32,35], as well as the formation of adsorption layers (“crowns”) of biopolymer molecules [33]. A certain role in Ni NPs’ action in vivo can also be played by various mechanisms of transport of these NPs through biological barriers, such as clathrin-dependent endocytosis and persorption [36]. However, to evaluate the significance of these putative mechanisms, additional data on the toxicokinetics of NiNPs are needed, which was not the aim of this study.

We should also consider the relatively high oral acute toxicity of metallic Ni NPs that we revealed in comparison with NiO NPs, which were characterized in [13] as non-toxic or extremely low toxic. The observed clinical picture of acute intoxication of Ni NPs, as the studies have shown, is based on their damaging effect on the peripheral nerve endings of the gastrointestinal tract with gross impairment of intestinal motility and evacuation. The fact that such phenomena were practically not observed for NiO NPs may be associated with the specific neurotoxic effect of Ni NPs, the mechanisms of which require a separate study.

An unexpected result of the present study was the absence of signs of increased apoptosis of liver cells in rats orally exposed to metal Ni NPs. The pro-apoptogenic effect of Ni-containing nanomaterials on cells is known from a large number of studies performed in vitro [37] and in vivo [28]. However, in the works presented in the available literature, apoptosis in the liver was observed mainly under the influence of NiO NPs, which differed from metallic Ni NPs both in biokinetic characteristics and main target organs and systems [29]. The absence of any effect of Ni NPs on the apoptosis of liver cells is consistent with the fact that, as a result of their oral administration, there is no accumulation of Ni in this organ (see Table 4), unlike pronounced kidney accumulation. The observed paradoxical decrease in the accumulation of total Ni in the liver tissue of rats fed with Ni NP1 and Ni NP2 cannot be satisfactorily explained based on the data obtained in this experiment. It may be assumed that this effect is associated with a decrease in the overall metabolic activity and the number of Ni-binding centers in the organ tissue due to the development of systemic intoxication accompanied by an inflammatory response and oxidative stress. The main site of accumulation of Ni, both in the case of exposure to its NPs as well as to its soluble salt, are the kidneys, which is consistent with the assumption that extra Ni is predominantly excreted in the urine.

## 5. Conclusions

Thus, nanoparticles of metallic Ni when orally administered to rats are characterized by the presence of signs of both acute and subchronic toxicity. Acute toxicity was higher for the preparation of Ni NP1 with a smaller average particle size, compared with the larger Ni NP2. By the value of LD_50_, the latter can be classified as substances of the IV hazard class, while the hazard class of Ni NP1 is suggested to be even higher. Most of the toxic effects manifested by Ni nanoforms were absent for the soluble salt of this element in comparable doses, which indicates the presence of metallic Ni nanotoxicity. The lowest observed effect level (LOEL) of metallic Ni NP1 was established at 10 mg/kg b.w. based on the endpoints, for which clear dependence “dose-effect” could be obtained. However, some effects of these NPs were noticed even at a dose of 0.1 mg/kg b.w. but were not confirmed at higher doses. The main targets of these NPs’ actions were the nervous and immune systems. Ni NP2 showed the presence of action at a dose of 0.1 mg/kg b.w. and higher, so their LOEL was established at a level less than 0.1 mg/kg, and the critical affected systems were also the brain and immune system. The influence of both types of Ni NPs on liver apoptosis was insignificant. To further clarify the toxicological characteristics of orally administered metallic Ni NPs, including their impact on different biological processes, it is planned to carry out studies using a wider set of specific biochemical, immunological, transcriptomic, and microbiome parameters.

## Figures and Tables

**Table 1:** Dimensional characteristics of Ni NP1 and Ni NP2 according to TEM data.

Dimensions, nm	Nanomaterials	
	Ni NP1	Ni NP2
Mean diameter (M)	53.7	70.9
Standard error of the mean (S.E.M.)	2.9	3.3
Standard deviation (S.D.)	29.3	32.0
Median (Med)	47.0	64.0
The smallest diameter	15.0	17.0
The largest diameter	180.0	192.0
The number of particles within dimensional range:		
Less than 50 nm	55.5	24.0
50–100 nm	37.8	57.1
More than 100 nm	6.7	18.9
Normality distribution test (Kolmogorov–Smirnov criterion), *p*	0.034	>0.1
ζ-potential, mV	+25.71	−3.33

**Table 2:** Mean (M ± S.E.M.) body weight and relative organ weight (% body weight) of rats at withdrawal from the experiment.

Group	Body Weight, g	Relative Organ Weight (% of Body Weight)			
		Liver	Spleen	Thymus	Brain
1. Control	413 ± 9	3.09 ± 0.06	0.28 ± 0.03	0.13 ± 0.01	0.46 ± 0.01
2. Ni S 0.1 mg/kg b.w.	415 ± 20	2.86 ± 0.11 *	0.31 ± 0.03	0.12 ± 0.01	0.50 ± 0.02
3. Ni S 1.0 mg/kg b.w.	431 ± 14	2.92 ± 0.13	0.28 ± 0.02	0.11 ± 0.01	0.48 ± 0.02
4. Ni S 10 mg/kg b.w.	407 ± 20	2.94 ± 0.07	0.21 ± 0.018	0.13 ± 0.01	0.51 ± 0.02
5. Ni NP1 0.1 mg/kg b.w.	384 ± 18	2.89 ± 0.08	0.21 ± 0.01 *^#^	0.13 ± 0.01	0.52 ± 0.03
6. Ni NP1 1.0 mg/kg b.w.	371 ± 14 *^#^	2.91 ± 0.07	0.29 ± 0.02	0.11 ± 0.01	0.53 ± 0.02 *
7. Ni NP1 10 mg/kg b.w.	407 ± 13	2.90 ± 0.09	0.30 ± 0.03	0.11 ± 0.01 *^#^	0.49 ± 0.01
8. Ni NP2 0.1 mg/kg b.w.	395 ± 7	2.80 ± 0.07*	0.22 ± 0.02 ^#^	0.10 ± 0.01 *^#^	0.51 ± 0.01 *
9. Ni NP2 1.0 mg/kg b.w.	401 ± 10	2.82 ± 0.05*	0.25 ± 0.02	0.11 ± 0.01 *	0.50 ± 0.01 *
10. Ni NP2 10 mg/kg b.w.	393 ± 14	2.80 ± 0.05*	0.21 ± 0.02 ^Ø^	0.11 ± 0.01 *	0.50 ± 0.01 *
Factors	-	Ni × nano	-	-	-

Notes: *—the difference with the control group is significant; ^#^—the difference with the group receiving soluble salt of Ni (Ni S) in an equivalent dose, is significant; ^Ø^—the difference with the group receiving Ni NP1 is significant; *p* < 0.05. Factors: distribution is not uniform, *p* < 0.05, 3-way ANOVA, by factors of Ni dose (Ni), presence of nanoform (nano). Data for kidney, adrenals, and testis are not presented (no significant differences noticed). The number of animals is 12 in each group.

**Table 3:** Hematological indicators (leucocytes and thrombocyte counts) in rats.

Group	Indicators (M ± S.E.M), Measurement Units							
	Leucocytes Total, 10^9^ × L^−1^	Neutro- Philes, 10^9^ × L^−1^	Basophiles, 10^7^ × L^−1^	Lympho- Cytes, 10^9^ × L^−1^	Monocytes, 10^9^ × L^−1^	Trombo- Cytes, 10^9^ × L^−1^	Mean Volume of Trombocyte, μm^3^	Trombocrit, %
1. Control	10.4 ± 1.0	1.73 ± 0.18	5.66 ± 0.78	6.92 ± 0.51	1.49 ± 0.38	634 ± 22	6.57 ± 0.15	0.42 ± 0.02
2. Ni S 0.1 mg/kg b.w.	7.5 ± 1.0	1.70 ± 0.39	4.65 ± 1.28	4.69 ± 0.66 *	0.87 ± 0.25	618 ± 41	6.84 ± 0.17	0.42 ± 0.03
3. Ni S 1.0 mg/kg b.w.	8.7 ± 1.5	1.84 ± 0.26	7.01 ± 1.99	5.46 ± 1.11	0.97 ± 0.22	622 ± 44	6.80 ± 0.24	0.42 ± 0.02
4. Ni S 10 mg/kg b.w.	7.8 ± 1.2	1.40 ± 0.36	4.35 ± 1.22	5.53 ± 0.90	0.95 ± 0.17	632 ± 39	6.54 ± 0.07	0.41 ± 0.02
5. Ni NP1 0.1 mg/kg b.w.	9.4 ± 1.5	1.72 ± 0.31	5.96 ± 1.74	7.06 ± 1.16	0.74 ± 0.11	648 ± 34	6.31 ± 0.11 ^#^	0.41 ± 0.02
6. Ni NP1 1.0 mg/kg b.w.	11.5 ± 1.3	2.85 ± 0.72	7.40 ± 1.42	7.72 ± 0.91	1.03 ± 0.13	515 ± 41	6.73 ± 0.16	0.34 ± 0.02 *^#^
7. Ni NP1 10 mg/kg b.w.	13.4 ± 1.6 ^#^	1.87 ± 0.16	7.48 ± 0.98	9.15 ± 1.09 ^#^	1.57 ± 0.12 ^#^	591 ± 41 ^#^	6.56 ± 0.12	0.39 ± 0.02
8. Ni NP2 0.1 mg/kg b.w.	12.0 ± 1.4 ^#^	2.42 ± 0.28	5.44 ± 0.89	6.98 ± 0.84 ^#^	1.27 ± 0.12 ^∅^	555 ± 30 ^∅^	6.54 ± 0.11	0.36 ± 0.02
9. Ni NP2 1.0 mg/kg b.w.	10.5 ± 0.7	1.95 ± 0.21	9.68 ± 0.90 *	7.14 ± 0.57	1.33 ± 0.17	544 ± 40	6.89 ± 0.32	0.37 ± 0.01
10. Ni NP2 10 mg/kg b.w.	11.5 ± 1.2	2.72 ± 0.41 ^#^	5.81 ± 0.97	7.47 ± 0.92	1.10 ± 0.14 ^∅^	578 ± 47 ^∅^	6.09 ± 0.09 *^#^^∅^	0.35 ± 0.03
Factors	nano			nano	Ni nano	-	Ni	nano

Notes: *—the difference with the control group is significant; ^#^—the difference with the group receiving soluble salt of Ni (Ni S) in equivalent dose, is significant; ^Ø^—the difference with the group receiving Ni NP1 is significant; *p* < 0.05. Factors: distribution is not uniform, *p* < 0.05, 3-way ANOVA, by factors of Ni dose (Ni), presence of nanoform (nano). Data for kidney, adrenals, and testis are not presented (no significant differences noticed). The number of animals is 8 in each group.

**Table 4:** Mean (M ± S.E.M.) Ni content in the organs of rats at withdrawal from the experiment.

Group	Content, μg/g (Wet Weight)		
	Liver	Kidney	Spleen
1. Control	1.12 ± 0.15	0.50 ± 0.04	0.39 ± 0.12
2. Ni S 0.1 mg/kg b.w.	1.35 ± 0.03	0.71 ± 0.25	0.20 ± 0.03
3. Ni S 1.0 mg/kg b.w.	1.65 ± 0.32	1.57 ± 0.28 *	0.18 ± 0.01
4. Ni S 10 mg/kg b.w.	0.76 ±0.06 *	3.94 ± 0.79 *	0.36 ± 0.04
5. Ni NP1 0.1 mg/kg b.w.	0.61 ± 0.05 *^#^	0.70 ± 0.21	0.24 ± 0.05
6. Ni NP1 1.0 mg/kg b.w.	0.61 ± 0.02 *^#^	0.65 ± 0.11 ^#^	0.15 ± 0.01
7. Ni NP1 10 mg/kg b.w.	0.66 ± 0.03 *	2.92 ± 0.46 *	0.33 ± 0.05
8. Ni NP2 0.1 mg/kg b.w.	0.62 ± 0.01 *	0.38 ± 0.04 *	0.16 ± 0.01
9. Ni NP2 1.0 mg/kg b.w.	0.58 ± 0.02 *^#^	0.68 ± 0.22 ^#^	0.16 ± 0.02
10. Ni NP2 10 mg/kg b.w.	0.58 ± 0.02 *^#Ø^	2.36 ± 0.55 *	0.22 ± 0.02 ^#^
Factors	Ni, nano, Ni×nano	Ni, nano	Ni

Notes: *—the difference with the control group is significant; #—the difference with the group receiving soluble salt of Ni (Ni S) in equivalent dose, is significant; ^Ø^—the difference with the group receiving Ni NP1 is significant; *p* < 0.05. Factors: distribution is not uniform, *p* < 0.05, 3-way ANOVA, by factors of Ni dose (Ni), presence of nanoform (nano), and combination thereof. The number of animals is 8 in each group.

## Data Availability

Data available on request due to restrictions, e.g., privacy or ethical.
